# MicroRNA (miR)-597-5p Inhibits Colon Cancer Cell Migration and Invasion by Targeting FOS-Like Antigen 2 (*FOSL2*)

**DOI:** 10.3389/fonc.2019.00495

**Published:** 2019-06-12

**Authors:** Shuo Li, Zhuo Liu, Xue-dong Fang, Xiu-ying Wang, Bing-yuan Fei

**Affiliations:** ^1^Department of Hepatobiliary and Pancreatic Surgery, China-Japan Union Hospital of Jilin University, Changchun, China; ^2^Department of Gastrointestinal Colorectal and Anal Surgery, China-Japan Union Hospital of Jilin University, Changchun, China; ^3^Medical Record Department, China Japan Union Hospital of Jilin University, Changchun, China

**Keywords:** colorectal cancer, CRC, *FOSL2*, miR-143-5p, FOS-like antigen 2

## Abstract

Our previous work had shown that FOS-like antigen 2 (*FOSL2*) is regulated by miR-143-5p in colorectal cancer (CRC). Given that it has been shown by others that *FOSL2* is also a target of miR-597-5p in breast adenocarcinoma, the objective of the current work was to determine whether *FOSL2* is regulated by miR-597-5p in CRC and the role of miR-597-5p in CRC. MiR-597-5p expression was determined in RNA obtained from 30 paired samples of colon cancer and tumor adjacent normal tissue, as well as in the LoVo (CRC cell line) and FHC (normal colonic epithelial cells) by quantitative real time polymerase chain reaction (qRT-PCR). MiR-597-5p expression was significantly downregulated in both CRC tissue and LoVo cells. Reporter assays using wild-type and miR-597-5p seed mutant *FOSL2* confirmed that *FOSL2* is a *bona fide* target of miR-597-5p. Modulating miR-597-5p expression levels in FHC and LoVo cells using antagomir and mimic, respectively, impacted expression of epithelial and mesenchymal cell markers as well as *in vitro* migration and invasion, without any effect on cell proliferation, showing that miR-597-5p functions as a suppressor of epithelial to mesenchymal transition. Restoration of *FOSL2* expression rescued pro-metastatic functional properties of LoVo cells conforming that effect of miR-597-5p was being mediated by targeting *FOSL2*. Xenograft assays in athymic nude mice showed that miR-597-5p mimic did not reduce tumor incidence or growth in LoVo cells. However, using a hepatic metastasis model showed that miR-597-5p mimic can significantly prevent hepatic metastatic nodule formation as well as *FOSL2* expression in these metastatic nodules. Importantly, *FOSL2* mRNA and miR-597-5p expression was found to be inversely correlated in an independent cohort of 21 CRC patients Cumulatively our results show that miR-597-5p functions as a suppressor of metastatic progression in CRC by targeting *FOSL2*. Replenishment of miR-597-5p can be a potential therapeutic target where its expression along with *FOSL2* can serve as potential diagnostic markers in CRC.

## Introduction

Development of chemotherapy resistance is responsible for majority of the fatalities in colorectal cancer (CRC), which contributes to ~9% of cancer mortality (1, 2). Surgical excision is the first line of treatment (3); however there is already onset of metastatic disease during diagnosis making surgical resection ineffective. Hence, additional markers for metastatic disease needs to be defined which can be potentially used for diagnosis and as therapeutic targets in CRC.

Sensitivity to pre-operative chemotherapy in CRC patients is linked to *KRAS* gene mutation (3, 4). Approximately 35–45% of CRC patients harbor mutations in *KRAS* (4–11), with exon 12 mutations accounting for 4/5th of total mutations (4). The response to neoadjuvant chemotherapy is poor when *KRAS* mutation is present. MicroRNAs (miRNAs) are non-coding RNAs that can bind target mRNA by complementary base pairing and inhibit translation of the mRNA. MiRNAs function as tumor suppressors and as oncogenes in CRC (12, 13). MiR-143-5p has been shown to target *KRAS* in CRC and prostate cancer (14, 15).

Previous work by us has shown that sensitivity of CRC cell lines to paclitaxel (PTX) treatment is dependent on *KRAS* mutation status and expression of miR-143-5p. LoVo cells that harbor *KRAS* mutation, but not SW480 cells with wild-type *KRAS*, were found to be sensitive to PTX treatment when miR-143-5p levels were restored by transfection of miR-143-5p mimic (16). Apart from this increased sensitivity, restoration of the miR-143-5p also caused inhibition of *in vitro* migration and invasion in LoVo cells (16), indicating that miR-143-5p regulates a pro-mesenchymal switch in these cells. Subsequent work by us showed that *FOSL2* was downregulated in LoVo cells when miR-143-5p was restored (17).

It has been recently shown that miR-597-5p targets *FOSL2* in normal breast epithelial cells and downregulation of miR-597-5p during breast cancer prognosis promote migratory behavior in breast cancer cells (18). Hence, the objective of the current study was to determine of miR-597-5p targets *FOSL2* in the context of CRC.

## Methods

### Patient Samples, Tissue Storage, Isolation of RNA, and Quantitative Real Time PCR (qRT-PCR)

The China Japan Union Hospital of Jilin University was the source of 30 paired samples (tumor samples from surgical resection and surrounding healthy tissues) collected retrospectively. Informed consent for using tissues for research was obtained from all enrolled patients. The Institutional Review Board of the China Japan Union Hospital approved the study. This study utilized samples from patients who did not display any co-morbid manifestations; tissues were subjected to snap freezing and liquid nitrogen storage. For the independent testing, paired samples were collected from 21 CRC patients undergoing surgical resection who did not have any comorbidities or did not undergo any neoadjuvant chemotherapy. Total RNA from the samples was isolated with TriZol as per instructions of the manufacturer (ThermoFisher Scientific, Shanghai, China). Quantitative RT-PCR was done using TaqMan miRNA or TaqMan gene expression probes (ThermoFisher Scientific) for *FOSL2*, miR-597-5p (Assay ID: 001551), and miR-143-5p (Assay ID: 002146) as described before (16). Internal controls were *RNU6B* (Assay ID: 001093) and *ACTB* (Assay ID: Hs03023943_g1) for data normalization for miRNA and mRNA expressions, respectively. –ΔΔCt method was utilized for data analysis that was expressed as mean ± standard deviation (SD).

### Cell Culture

LoVo (mutant *KRAS*—G13D, A14V, wild-type *BRAF, PIK3CA, PTEN*, and *TP53*) (19) and the FHC (normal colonic epithelial cells) were obtained from the ATCC (Manassas, VA, USA) kept in a CO_2_ incubator at 37°C. Dulbecco's modified Eagle's media (DMEM) containing 10% fetal bovine serum (FBS) and penicillin-streptomycin (1%) was used to culture LoVo cells. The FHC was cultured using in DMEM/F12 media containing 10% FBS, 10 ng/ml cholera toxin, 10 mM HEPES, insulin and transferrin both at 0.005 mg/ml plus 100 ng/ml hydrocortisone.

### Reporter and Expression Constructs

The 3′ UTR of the endogenous *FOSL2* was amplified from genomic DNA using 5′- gtcctcctcgctcctcctt−3′ and 5′–tgctactcaactgaaagtggaaa−3′ forward and reverse primer, respectively. The amplified 4,869 bp product was cloned into pRL vector (Promega) to construct the 3′ UTR reporter. Mutant construct of *FOSL2* 3′UTR was generated using deletion of the site that binds miR-597-5p seed region using site-directed mutagenesis using QuickChange II kit (Agilent) and the following primers: 5′-ccccgtggagaaagcaattcacacagctgttc-3′ and 5′-gaacagctgtgtgaattgctttctccacgggg-3′. Assays involving luciferase involved a control for transfection as well as normalization with a firefly luciferase vector (Promega). UCSC human genome reference version hg19 was used to verify sequences of all the constructs. pcDNA3-*FOSL2* overexpression plasmid was described before (16).

### Cell Transfection and Luciferase Assay

MiR-597-5p mimic and anti-miR-597-5p antagomir were obtained from ThermoFisher Scientific. 50 nM of the mimic or antagomir, or luciferase reporter plasmids was used for transient transfection of FHC or LoVo as shown using Lipofectamine LTX in accordance to an earlier protocol (16). Luciferase assay was performed with the Dual Luciferase Assay kit (Promega). The expression of Renilla luciferase was subjected to normalization with respect to expression of Firefly luciferase. Mean ± standard deviation (SD) was used to represent data of three independent experiments.

### Preparation of Whole Cell Lysates and Immunoblot Analysis

Immunoblot analysis was done as described previously (16). Blots were probed with the following antibodies as indicated: E-cadherin (clone 4A2; catalog # 231303), Fibronectin (catalog # 2413), Vimentin (catalog # 24525), GAPDH (catalog # 9485) (Abcam, Cambridge, MA, USA), and *FOSL2* (catalog # LS-C116891, Seattle, WA, USA).

### MTT Assay for Cell Proliferation

Ninety-six well plates with clear bottom were used to culture cells. An assay based on colorimetry (MTT assay, Sigma-Aldrich, St. Louis, MO, USA) was used to determine cell proliferation according to manufacturer guidelines. Absorbance value at 570 nm was subjected to correction by using absorbance at 690 nm (reference) that was subtracted from the former. The relative corrected absorbance was used to determine relative proliferation, Data is represented as mean ± SD.

### Transwell Migration and Invasion Assays

CytoSelect 24-Well Cell Migration and Invasion Assay (8 μm) (Cell BioLabs, San Diego, CA, USA) was utilized for these assays in accordance to instructions of the manufacturer. Images were obtained at 10X magnification. Data obtained were used to analyze percent migration and invasion and were expressed as mean ± standard deviation.

### Xenograft Assay

All animal experiments reported in the current study was approved by the Institutional Animal Care and Use Committee of China Japan Union Hospital of Jilin University. Xenograft assay was performed as described previously (17). For xenograft assays, 10^6^ LoVo cells, subcutaneously injected into 8 weeks old nude mice (*n* = 12). Half of the animals received weekly injections of miR-597-5p mimic (13 μg/week in 100 μl volume) along with an equal volume of Lipofectamine 2000 at the original injection site. The *mock* group received equal volume of phosphate buffered saline instead of the miR-597-5p mimic. Tumor diameters were measured every 5 days, and volumes calculated using the estimation: width^2^ × length × 0.5. Animals were sacrificed on day 42 and tumor weight was measured off excised tumors. To measure metastatic potential, 5 × 10^5^ LoVo cells were injected into the right lower lobe of the liver in mice (*n* = 16) as described before (17). For half of the animals, miR-597-5p mimic (13 μg/week in 100 μl volume) were administered by tail-vein injection, using equal volume mixtures of Lipofectamine 2000. The other *mock* group received equal volume of phosphate buffered saline instead of the miR-597-5p mimic. Animals were sacrificed on day 28 and all metastatic nodules were counted in the left lobe and data represented as mean ± SD. The liver tissues were carefully excised and fixed with 10% formalin solution. Paraffin sections (5 μm thickness) were prepared for hematoxylin-eosin (H&E) staining and immunohistochemistry (IHC) staining using anti-*FOSL2* antibody using DAB chromogenic method.

### Statistical Analyses

Mean ± SD is the mode of expressing all the quantitative data unless specified otherwise. ANOVA with Tukey's *post-hoc* test was used to measure difference between groups using Origin Pro (OriginLab Corporation) with a significance level of ^*^P < 0.05.

## Results

**Figure 1:**
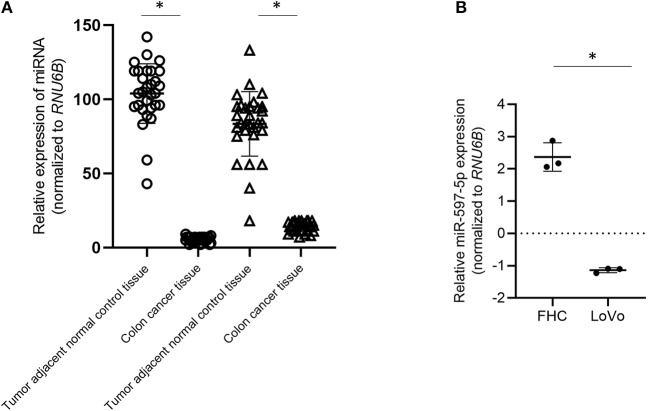
Down regulation of MiR-597-5p in colon cancer cells. **(A)** qRT-PCR analysis of miR-597-5p and miR-143-5p using RNA isolated from 30 paired cancer and tumor adjacent normal colon tissue specimens. **(B)** qRT-PCR analysis of miR-597-5p in LoVo and FHC cells (normal colonic epithelium). Data in **(A,B)** were normalized based on *RNU6B* expression. Data expressed as mean ± SD of three independent experiments. *P < 0.05.

All patient samples collected were from Stage II and III CRC patients who had undergone surgical resection and did not have any co-morbidities. We initially determined the expression of miR-597-5p and miR-143-5p expression levels in these 30 paired samples. Expression of both miR-143-5p and miR-597-5p were downregulated compared to tumor adjacent normal tissue (*P* < 0.05 in each case; Figure 1A). We did not find any significant correlation of miR-597-5p expression and clinicopathological features, inclusive of age, gender, disease stage, and survival (*data not shown*). This can potentially be attributed to the small sample size of our study cohort. Whether miR-143-5p and miR-597-5p have redundant or overlapping roles in preventing colon cancer remains to be evaluated.

We next determined the relative expression of miR-597-5p in the colon cancer cell line LoVo and the normal colonic epithelium cell line FHC cells. Similar to what was observed in CRC patient samples, expression of miR-597-5p was significantly down regulated in the LoVo cells in comparison to that of the FHC cells (*P* < 0.05; Figure 1B). Cumulatively, these results indicated that the expression of miR-597-5p is downregulated in colon cancer.

**Figure 2:**
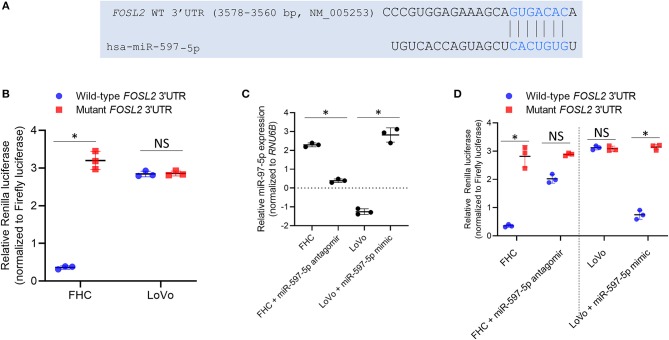
*FOSL2* is targeted by miR-597-5p in colon cells. **(A)** Schematic representation of complementarity of *FOSL2* 3′ UTR and miR-597-5p seed sequence. **(B)** Relative Renilla luciferase activity of constructs with wild type 3′ UTR of *FOSL2* or its mutant (with deletion of miR-597-5p binding site) in FHC and LoVo cells. **(C)** QRT-PCR analysis of miR-597-5p in FHC and LoVo cells transfected with miR-597-5p antagomir and mimic, respectively. **(D)** Relative Renilla luciferase activity of constructs with wild type or mutant *FOSL2* 3′ UTR of in FHC and LoVo cells, alone or in combination with anti-miR-597-5p antagomir or miR-597-5p mimic, respectively. All data in **(B–D)** are expressed as mean ± SD of three independent experiments. *P < 0.05; NS, non-significant.

We next assessed whether *FOSL2* is a true target of miR-597-5p in the context of colon cancer. FHC and LoVo cell lines were transfected using Renilla luciferase reporter plasmids harboring either wild-type *FOSL2* 3′ UTR or miR-597-5p seed mutant 3′ UTR (Figure 2A). Renilla luciferase expression was significantly inhibited in the FHC cells compared to the LoVo cells (2.5 ± 0.05 fold; *P* = 0.006) (Figure 2B). Of note, miR-597-5p expression is higher in FHC cells compared to the LoVo cells (Figure 1B).

In order to confirm that the observed differential luciferase activity of the wild-type reporter was caused by targeting of the *FOSL2* 3′ UTR by miR-597-5p in the FHC cells, a miR-597-5p seed mutant *FOSL2* reporter plasmid was generated and tested. There was no statistically significant difference between the LoVo and FHC cells in the case of the mutant construct (*P* > 0.05; Figure 2B). This indicated that *FOSL2* is a potential target of miR-597-5p. To further confirm this observation, FHC cells were transfected with anti-miR-597-5p antagomir and LoVo cells were transfected with miR-597-5p mimic. Successful overexpression and downregulation of miR-597-5p in LoVo and FHC cells, respectively, were verified by qRT-PCR (Figure 2C). Transfection of miR-597-5p antagomir in FHC cells significantly de-repressed Renilla luciferase expression, whereas miR-597-5p mimic transfection in LoVo cells significantly attenuated Renilla luciferase expression (Figure 2D; *P* < 0.05 in each case compared to mock transfection). This confirmed that *FOSL2* is a *bona fide* target of miR-597-5p in this context. Collectively, these observations are a confirmation that miR-597-5p targets *FOSL2* in normal colonic epithelial cells and that its down regulation in tumorigenic colon cells results in the induction of *FOSL2* expression in colon cancer cells.

**Figure 3:**
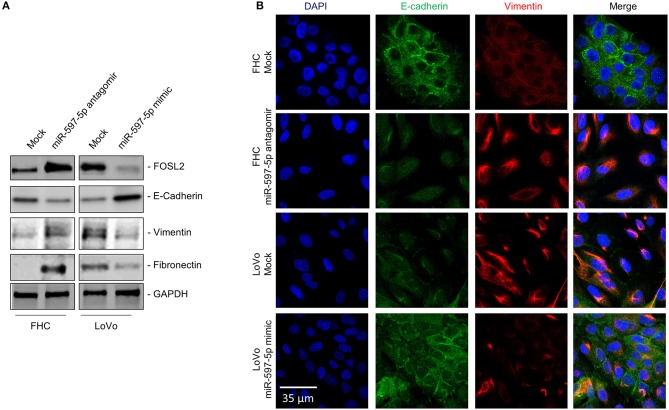
MiR-597-5p replenishment in the LoVo cells downregulates mesenchymal cell markers. **(A)** Immunoblot analysis to determine the effect of miR-597-5p replenishment in LoVo cells and inhibition in the FHC cells on *FOSL2* and epithelial (E-Cadherin) and mesenchymal cell (Fibronectin and Vimentin) markers. Following stripping of blots, GAPDH was used to probe for equal loading of samples. From among three independent experiments, one representative blot is shown here. **(B)** Representative immunofluorescence images of E-cadherin and Vimentin in LoVo cells and FHC cells, mock transfected or co-transfected with miR-597-5p mimic and antagomir, respectively. Scale bar, 30 μm.

We next determined expression of *FOSL2* and epithelial (E-cadherin) and mesenchymal cell (Vimentin and Fibronectin) markers in FHC and LoVo cells transfected with miR-597-5p antagomir and mimic, respectively. MiR-597-5p antagomir increased *FOSL2* expression in FHC cells (Figure 3A) and induced mesenchymal cell markers and downregulated E-cadherin (Figures 3A,B). Conversely, miR-597-5p mimic decreased *FOSL2* protein expression in LoVo cells (Figure 3A) and induced E-cadherin, while suppressing Fibronectin and Vimentin (Figures 3A,B). Cumulatively, these results suggest that miR-597-5p inhibits epithelial to mesenchymal transition (EMT) in normal colon epithelial cells and its downregulation in CRC cells promotes EMT.

**Figure 4:**
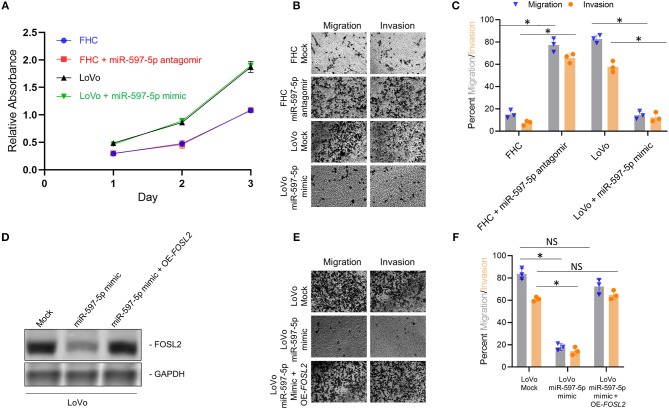
Manipulation of miR-597-5p expression affects *in vitro* migration and invasion via *FOSL2*. **(A)** Proliferation of indicated cells was examined with MTT assay. Every experiment was conducted for a minimum of three times. **(B,C)** Representative images **(B)** and quantification **(C)** of *in vitro* migration and invasion, in indicated cell variants; *P < 0.05; NS, non-significant. **(D)** Immunoblot analysis of *FOSL2* in mock transfected LoVo cells, and LoVo cells transfected with miR-597-5p mimic alone or in combination with expression plasmid encoding *FOSL2*. GAPDH was used as a loading control. Shown are representative blots from three experiments. **(E**,**F)** Representative images **(E)** and quantification (**F**; *P < 0.05; NS, non-significant) of *in vitro* migration and invasion, in mock transfected LoVo cells, and in LoVo cells transfected with miR-597-5p mimic alone or in combination with expression plasmid encoding *FOSL2*. Data in **(A,C,E)** are represented as mean ± SD.

However, effect of modulating miR-597-5p on *FOSL2* expression and EMT in FHC and LoVo cells were independent of any significant changes in proliferation (Figure 4A), indicating that miR-597-5p exclusively suppresses pro-metastatic pathways in CRC cells. To confirm the same, we performed *in vitro* migration and invasion assay, two prominent functional readouts of mesenchymal cells. Mir-597-5p antagomir significantly increased *in vitro* migration and invasion in FHC cells (Figures 4B,C; *P* < 0.05 in each case compared to mock transfection). Conversely, miR-597-5p antagomir significantly inhibited *in vitro* migration and invasion in the LoVo cells (Figures 4B,C; *P* < 0.05 in each case compared to mock transfection). These results cumulatively confirmed that expression of miR-597-5p has an inverse correlation with expression of EMT marker expression as well as invasion and migration of CRC cells *in vitro*.

MiRNAs have multiple mRNA targets. Hence, to determine if the effect of modulating miR-597-5p expression on EMT and *in vitro* migration and invasion is being mediated via *FOSL2*, we performed rescue experiment in the LoVo cells. LoVo cells transfected with miR-597-5p mimic were co-transfected with *FOSL2* expression plasmid lacking the 3′ UTR. Successful overexpression of *FOSL2* protein expression was confirmed by western blot (Figure 4D). Next, we determined the effect of *FOSL2* overexpression in LoVo cells transfected with miR-507-5p mimic on *in vitro* migration and invasion. As shown in Figures 4B,C, miR-597-5p transfection in LoVo cells significantly downregulated *in vitro* migration and invasion (Figures 4D–F). However, overexpression of *FOSL2* rescued both *in vitro* migration and invasion capacity in LoVo cells transfected with miR-597-5p (Figures 4D–F), confirming that miR-597-5p is regulating EMT and *in vitro* migration and invasion via targeting *FOSL2*.

**Figure 5:**
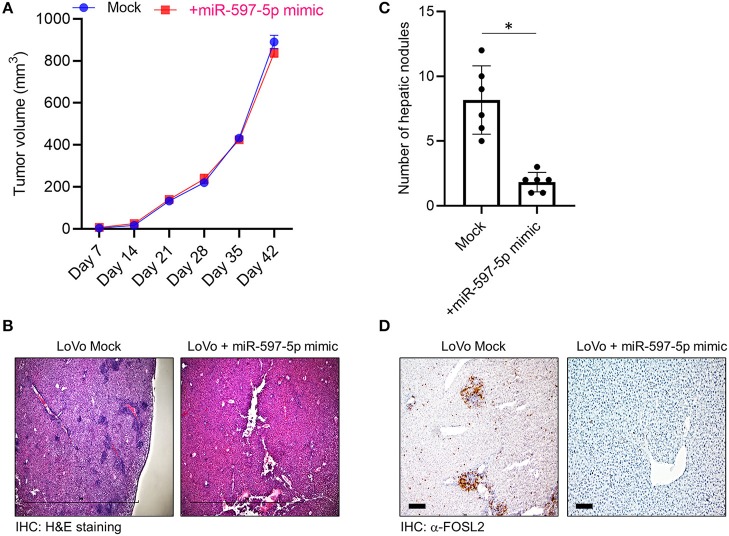
MiR-597-5p can suppress metastatic hepatic burden but not tumor growth rate *in vivo*. **(A)** Restoration of miR-597-5p expression by mimic did not affect tumor growth as assayed in a subcutaneous tumor model (*n* = 12). **(B,C)** Mice in the miR-597-5p mimic group, compared to the mock group, had significant less hepatic tumor burden as assayed by the presence of hepatic nodules in non-injected liver lobes. **(B)** shows representative H&E staining (scale bar, 1 mm) and **(C)** is quantification of **(B)** (*n* = 8 mice/group; *P < 0.05; NS, non-significant). **(D)** IHC analysis of *FOSL2* in non-injected hepatic lobes showed downregulation of *FOSL2* expression and hepatic nodes in mice getting tail vein injection of miR-597-5p mimic. Representative images from six mice are shown. Scale bar, 10 μm.

To determine if the *in vitro* effect of miR-597-5p modulation on *in vitro* migration and invasion in LoVo cells is operant *in vivo*, we performed xenograft assays. LoVo cells were subcutaneously injected into athymic nude mice. Half of the animals received weekly injection of miR-597-5p mimic at the original injection sire. Tumor formation and growth was monitored. Both experimental groups formed tumors within 12 days but there was no significant difference in tumor volume after 42 days (Figure 5A), indicating that modulating miR-597-5p expression perhaps do not affect *in vivo* proliferation of LoVo cells. To determine if miR-597-5p mimic will affect *in vivo* metastasis, we used a hepatic tumor growth model. MiR-597-5p mimic significantly inhibited hepatic metastasis (Figure 5B; *P* < 0.05 compared to the no-mimic group) and *FOSL2* expression in the non-injected liver lobes (Figures 5C,D). These results confirm that miR-597-5p is an inhibitor of metastatic progression of CRC and its downregulation promotes CRC metastasis, and that this effect is mediated by miR-597-5p-mediated regulation of *FOSL2* protein expression.

**Figure 6:**
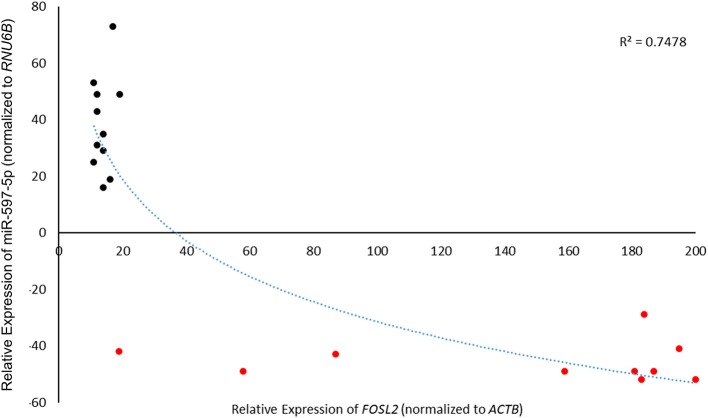
MiR-597-5p and *FOSL2* mRNA expression show inverse correlation in colorectal cancer patients. Pearson correlation of 21 paired CRC tumor tissue and adjacent normal tissue was used to check correlation between *FOSL2* and miR-597-5p. Expression was determined by qRT-PCR as described in Methods section and Figure 1 legend.

Given that our current experiments and earlier results (17) indicated that miR-597-5p and *FOSL2* have an inverse effect on EMT markers and *in vitro* motility in LoVo cells, we put forth a hypothesis that human colon cancer may have suppressed levels of miR-597-5p leading to *FOSL2* upregulation. In order to study the association between miR-597-5p and *FOSL2* in human colon cancer, we checked the expression of miR-597-5p and *FOSL2* in an independent cohort of 21 CRC patients. We observed an inverse correlation between up regulation of *FOSL2* levels and decreased miR-597-5p levels (*R*^2^ = 0.7478; Figure 6).

## Discussion

The expression of FOS and JUN isoforms is regulated by FOS-like antigen 2 (*FOSL2*: another name is FRA-2), FRA-2, a member of AP-1 transcription factor family (20). This protein is also crucially involved in cancer, fibrosis, differentiation of T cells, development of bones, and circadian rhythm (21–26). Another point of interest is the potential regulation of TGF-beta signaling pathway by *FOSL2* (27). This pathway is involved actively in metastasis of colon cancer (28) as well as, preservation of the stem cell niche inside the tumor (29), that in turn contributes to adjuvant chemotherapy resistance. This could account for the direct effect of *FOSL2* expression on motility and *in vivo* metastasis of the LoVo cells. Resistance to chemotherapy shows an intimate association with epithelial to mesenchymal transition (30, 31). As *FOSL2* favors a mesenchymal phenotype, it is not unexpected that the expression of this factor can influence the sensitivity of colon cancer cells to PTX (16).

An engaging point is that LoVo cells that were transfected with miR-143-5p mimic showed downregulated levels of solely *FOSL2* among 10 putative targets. The role of involvements of other gene(s) in resistance or sensitivity to PTX should be determined by studying tall the RNA molecules that are expressed differentially in the presence of miR-143-5p mimic. The effect of expression of *FOSL2* and colon cancer cells possessing wild-type *KRAS* in terms of sensitivity is yet to be studied. As migration and invasion of epithelial cells of the human colon (FHC cell line) *in vitro* was influenced by changing levels of *FOSL2*, we can hypothesize that regardless of the *KRAS* (wild-type or mutant), the sensitivity of colon cancer cells is influenced by the expression of *FOSL2*.

Role of miRNAs in the pathogenesis of different cancers is being increasingly appreciated. However, to the best of our knowledge, this study is a first that shows differential regulation of *FOSL2* by miR-597-5p in normal and tumorigenic colon cancer cells. It has been earlier shown that miR-597-5p is downregulated in breast cancer patients and targets *FOSL2* in normal breast epithelial cells (18). It has been shown that miR-597-5p is downregulated in epithelial ovarian cancer (32) and glioblastoma (33). It has also been shown that replenishment of miR-597-5p in ovarian cancer cells sensitizes them to cisplatin treatment by targeting *ABCB1* (34). Whether *FOSL2* are also being targeted by miR-597-5p in normal ovarian or brain cell remains to be determined.

Our earlier work has shown that miR-143-5p replenishment in LoVo cells sensitizes them to PTX treatment by targeting *FOSL2* (16). This represents a unique regulatory role of the same gene required for metastatic progression by multiple miRNAs. The regulation of *FOSL2* in the context of breast cancer (18) and *ABCB1* in the context of ovarian cancer (34) by miR-143-5p is yet to be studied. It will be important to establish if concomitant downregulated levels of *FOSL2* along with replenishment of miR-597-5p will synergize and chemosensitizes CRC cells to PTX-mediated cytotoxicity.

## Ethics Statement

Informed consent for using tissues for research duly signed from the patients was collected. The Institutional Review Board of the Hospital gave approval for the study.

## Author Contributions

SL designed the experiment. ZL and XF performed the experiments and analyzed the data. XW and BF prepared the manuscript. All authors have read the manuscript.

### Conflict of Interest Statement

The authors declare that the research was conducted in the absence of any commercial or financial relationships that could be construed as a potential conflict of interest.

## References

[1] Fidler IJ. Critical factors in the biology of human cancer metastasis: twenty-eighth G.H.A. Clowes memorial award lecture. Cancer Res. (1990) 50:6130–8. 1698118

[2] Ali M Kenneth K Burcu C Cynthia XM Targeted therapy for breast cancer. Front Oncol. (2013) 3:1096–112. 10.3389/fonc.2013.00250

[3] Chang GJ Kaiser AM Mills S Rafferty JF Buie WD. Practice parameters for the management of colon cancer. Dis Colon Rectum. (2012) 55:831–43. 10.1097/DCR.0b013e3182567e13 22810468

[4] Cong T Xiang D KRAS mutation testing in metastatic colorectal cancer. World J Gastroenterol. (2012) 18:5171–80. 10.3748/wjg.v18.i37.5171 23066310 PMC3468848

[5] Amado R Wolf M Peeters M Van-Cutsem E Siena S Freeman D. Wild-type KRAS is required for panitumumab efficacy in patients with metastatic colorectal cancer. J Clin Oncol. (2008) 26:1626–34. 10.1200/JCO.2007.14.7116 18316791

[6] Carsten B Igor B Anatoly M Hartmann JT Jorge A Filippo DB Fluorouracil, leucovorin, and oxaliplatin with and without cetuximab in the first-line treatment of metastatic colorectal cancer. J Clin Oncol. (2009) 27:663–71. 10.1200/JCO.2008.20.8397 19114683

[7] Eric VC Claus-Henning KH Erika H Jerzy Z Chung-Rong CC Anatoly M Cetuximab and chemotherapy as initial treatment for metastatic colorectal cancer. N Engl J Med. (2009) 360:1408–17. 10.1056/NEJMoa0805019 19339720

[8] Jean-Yves D Salvatore S James C Josep T Ronald B Mario B Randomized, phase III trial of panitumumab with infusional fluorouracil, leucovorin, and oxaliplatin (FOLFOX4) versus FOLFOX4 alone as first-line treatment in patients with previously untreated metastatic colorectal cancer: the PRIME study. J Clin Oncol. (2010) 28:4697–705. 10.1200/JCO.2009.27.4860 20921465

[9] Jochen G Marian G Klaus J Markus S Peter J Christoph O KRAS and BRAF mutations in patients with rectal cancer treated with preoperative chemoradiotherapy. Radiother Oncol. (2010) 94:76–81. 10.1016/j.radonc.2009.10.001 19913317 PMC7373270

[10] Peeters M Price TJ Cervantes A Sobrero AF Ducreux M Hotko Y. Randomized phase III study of panitumumab with fluorouracil, leucovorin, and irinotecan (FOLFIRI) compared with FOLFIRI alone as second-line treatment in patients with metastatic colorectal cancer. J Clin Oncol. (2010) 28:4706–13. 10.1200/JCO.2009.27.6055. 20921462

[11] Eric VC Claus-Henning KH István L Gunnar F Nowacki MP Stefano C Cetuximab plus irinotecan, fluorouracil, and leucovorin as first-line treatment for metastatic colorectal cancer: updated analysis of overall survival according to tumor KRAS and BRAF mutation status. J Clin Oncol. (2011) 29:2011–9. 10.1200/JCO.2010.33.5091 21502544

[12] Card DA Hebbar PB Li L Trotter KW Komatsu Y Mishina Y. Oct4/Sox2-regulated miR-302 targets cyclin D1 in human embryonic stem cells. Mol Cell Biol. (2008) 28:6426–38. 10.1128/MCB.00359-08 18710938 PMC2577422

[13] Hu S Wilson KD Ghosh Z Han L Wang Y Lan F. MicroRNA-302 increases reprogramming efficiency via repression of NR2F2. Stem Cells. (2013) 31:259–68. 10.1002/stem.1278 23136034 PMC3572288

[14] Chen L Liu H Li H Hsueh C Yu J Liang C. Thymidine phosphorylase mRNA stability and protein levels are increased through ERK-mediated cytoplasmic accumulation of hnRNP K in nasopharyngeal carcinoma cells. Oncogene. (2009) 28:1904–15. 10.1038/onc.2009.55 19330019

[15] Xu B Niu X Zhang X Tao J Wu D Wang Z miR-143-5p decreases prostate cancer cells proliferation and migration and enhances their sensitivity to docetaxel through suppression of KRAS. Mol Cell Biochem. (2011) 350:207–13. 10.1007/s11010-010-0700-6 21197560

[16] Fei BY Wang XY Fang XD MicroRNA-143 replenishment re-sensitizes colorectal cancer cells harboring mutant, but not wild-type, KRAS to paclitaxel treatment. Tumor Biol. (2016) 37:5829–35. 10.1007/s13277-015-4354-6 26581910

[17] Li S Fang XD Wang XY Fei BY. Fos-like antigen 2 (FOSL2) promotes metastasis in colon cancer. Exp Cell Res. (2018) 373:57–61. 10.1016/j.yexcr.2018.08.016 30114390

[18] He J Mai J Li Y Chen L Xu H Zhu X miR-597-5p inhibits breast cancer cell proliferation, migration and invasion through *FOSL2*. Oncol Rep. (2017) 37:2672–8. 10.3892/or.2017.5558 28393251 PMC5428280

[19] Ahmed D Eide PW Eilertsen IA Danielsen SA Eknæs M Hektoen M. Epigenetic and genetic features of 24 colon cancer cell lines. Oncogenesis. (2013) 2:e71. 10.1038/oncsis.2013.35 24042735 PMC3816225

[20] Tulchinsky E. Fos family members: regulation, structure and role in oncogenic transformation. Histol Histopathol. (2000) 15:921–8. 10.14670/HH-15.921 10963134

[21] Karin ML Heike RD Birte A Bahriye A Gabriele H Anja B The role of the AP-1 transcription factors c-Fos, FosB, Fra-1 and Fra-2 in the invasion process of mammary carcinomas. Breast Cancer Res Treat. (2004) 86:139–52. 10.1023/B:BREA.0000032982.49024.71 15319566

[22] Engel L Gupta BB Lorenzkowski V Heinrich B Schwerdtle I Gerhold S. Fos-related antigen 2 (Fra-2) memorizes photoperiod in the rat pineal gland. Neuroscience. (2005) 132:511–8. 10.1016/j.neuroscience.2004.12.014 15802201

[23] Roy S Khanna SA Schnitt R He G Weigert C Ichijo H. Fra-2 mediates oxygen-sensitive induction of transforming growth factor beta in cardiac fibroblasts. Cardiovasc Res. (2010) 87:647–55. 10.1093/cvr/cvq123 20427335 PMC2920807

[24] Ciofani M Madar A Galan C Sellars ML Mace K Pauli F. A validated regulatory network for Th17 cell specification. Cell. (2012) 151:289–303. 10.1016/j.cell.2012.09.016 23021777 PMC3503487

[25] Aline B Latifa B Maria J Rosen ED Philip CL Thorsten S Osteoblast-specific expression of Fra-2/AP-1 controls adiponectin and osteocalcin expression and affects metabolism. J Cell Sci. (2013) 126:5432–40. 10.1242/jcs.134510 24046454

[26] Zhou L Graves M Macdonald G Cipollone J Mueller CR Roskelley CD. Microenvironmental regulation of BRCA1 gene expression by c-Jun and Fra2 in premalignant human ovarian surface epithelial cells. Mol. Cancer Res. (2013) 11:272–81. 10.1158/1541-7786.MCR-12-0395 23339184

[27] Schröder C Schumacher U Müller V. The transcription factor Fra-2 promotes mammary tumour progression by changing the adhesive properties of breast cancer cells. Eur J Cancer. (2010) 46:1650–60. 10.1016/j.ejca.2010.02.008 20226654

[28] Chruścik A Gopalan V Lam AK. The clinical and biological roles of transforming growth factor beta in colon cancer stem cells: a systematic review. Eur J Cell Biol. (2017) 97:15–22. 10.1016/j.ejcb.2017.11.001 29128131

[29] Villalba M Evans SR Vidal-Vanaclocha F Calvo A. Role of TGF-β in metastatic colon cancer: it is finally time for targeted therapy. Cell Tissue Res. (2017) 370:29–39. 10.1007/s00441-017-2633-9 28560691

[30] Nieto MA Huang RYJ Jackson R Thiery JP. EMT: 2016. Cell. (2016) 166:21–45. 10.1016/j.cell.2016.06.028 27368099

[31] Brabletz T Kalluri R Nieto MA Weinberg RA. EMT in cancer. Nat Rev Cancer. (2018) 18:128–34. 10.1038/nrc.2017.118 29326430

[32] Zhou QH Zhao YM Jia LL Zhang Y. Mir-595 is a significant indicator of poor patient prognosis in epithelial ovarian cancer. Eur Rev Med Pharmacol Sci. (2017) 4278–82. 29077170

[33] Hao Y Zhang S Sun S Zhu J Xiao Y. MiR-595 targeting regulation of SOX7 expression promoted cell proliferation of human glioblastoma. Biomed Pharmacother. (2016) 80:121–6. 10.1016/j.biopha.2016.03.008 27133048

[34] Tian S Zhang M Chen X Liu Y Lou G. MicroRNA-595 sensitizes ovarian cancer cells to cisplatin by targeting ABCB1. Oncotarget. (2016) 7:87091. 10.18632/oncotarget.13526 27893429 PMC5349973

